# Shotgun sequencing of sonication fluid for the diagnosis of orthopaedic implant-associated infections with *Cutibacterium acnes* as suspected causative agent

**DOI:** 10.3389/fcimb.2023.1165017

**Published:** 2023-05-17

**Authors:** Diana Salomi Ponraj, Michael Lund, Jeppe Lange, Anja Poehlein, Axel Himmelbach, Thomas Falstie-Jensen, Nis Pedersen Jørgensen, Christen Ravn, Holger Brüggemann

**Affiliations:** ^1^ Department of Clinical Medicine, Aarhus University, Aarhus, Denmark; ^2^ Department of Biomedicine, Aarhus University, Aarhus, Denmark; ^3^ Department of Orthopaedic Surgery, Regional Hospital, Horsens, Denmark; ^4^ Department of Genomic and Applied Microbiology, Institute of Microbiology and Genetics, University of Göttingen, Göttingen, Germany; ^5^ Leibniz Institute of Plant Genetics and Crop Plant Research (IPK), Gatersleben, Germany; ^6^ Department of Orthopaedic Surgery, Aarhus University Hospital, Aarhus, Denmark; ^7^ Department of Infectious Diseases, Aarhus University Hospital, Aarhus, Denmark

**Keywords:** *Cutibacterium acnes*, shotgun sequencing, metagenomics, orthopaedic implant-associated infections, prosthetic infections, sonication fluid

## Abstract

Orthopaedic implant-associated infections (OIAIs) due to *Cutibacterium acnes* can be difficult to diagnose. The aim of this pilot study was to determine if metagenomic next-generation sequencing (mNGS) can provide additional information to improve the diagnosis of *C. acnes* OIAIs. mNGS was performed on sonication fluid (SF) specimens derived from 24 implants. These were divided into three groups, based on culture results: group I, culture-negative (n = 4); group II, culture-positive for *C. acnes* (n = 10); and group III, culture-positive for other bacteria (n = 10). In group I, sequence reads from *C. acnes* were detected in only one SF sample, originating from a suspected case of OIAIs, which was SF and tissue culture-negative. In group II, *C. acnes* sequences were detected in 7/10 samples. In group III, *C. acnes* sequence reads were found in 5/10 samples, in addition to sequence reads that matched the bacterial species identified by culture. These samples could represent polymicrobial infections that were missed by culture. Taken together, mNGS was able to detect *C. acnes* DNA in more samples compared to culture and could be used to identify cases of suspected *C. acnes* OIAIs, in particular regarding possible polymicrobial infections, where the growth of *C. acnes* might be compromised due to a fast-growing bacterial species. However, since SF specimens are usually low-biomass samples, mNGS is prone to DNA contamination, possibly introduced during DNA extraction or sequencing procedures. Thus, it is advisable to set a sequence read count threshold, taking into account project- and NGS-specific criteria.

## 1 Introduction

*Cutibacterium acnes* (formerly known as *Propionibacterium acnes*) is a Gram-positive slow-growing anaerobic bacterium (SGAB). It is a skin commensal that has also been implicated in various implant-associated infections, including orthopaedic implant-associated infections (OIAIs) (Zeller et al., 2007; McDowell et al., 2013; Achermann et al., 2014; Aubin et al., 2014; Ponraj et al., 2021). However, the diagnosis of OIAIs caused by SGAB such as *C. acnes* is complicated because of non-specific or even absent clinical, radiological, histopathological, and laboratory diagnostic features (Achermann et al., 2014; Aubin et al., 2014; Ponraj et al., 2021). Microbiological diagnosis based on culture can also be constrained by the need for prolonged incubation, due to the bacterium’s slow-growing nature, along with the attendant increased risk of sample contamination (Butler-Wu et al., 2011).

Culture-independent methods can potentially help to offset this problem (Gu et al., 2019). Culture-independent microbiological methods contain polymerase chain reaction (PCR)-based methods and sequencing-based methods; the latter comprise amplicon-based next-generation sequencing (aNGS) and metagenomic NGS (mNGS) (Tande and Patel, 2014; Ponraj et al., 2021). The utility of mNGS in the diagnosis of OIAIs from various specimens including synovial fluid aspirates and tissue samples has been reported previously (Ivy et al., 2018; Weaver et al., 2019; Cai et al., 2020; Fang et al., 2020; Huang et al., 2020; Kildow et al., 2021; Tan et al., 2022). However, only a few mNGS studies have so far been reported regarding the use of sonication fluid (SF) to support the diagnosis of OIAIs (Street et al., 2017; He et al., 2021) and, to the best of our knowledge, none with a specific focus on *C. acnes*.

In our previous study, we reported on the sonication of 100 orthopaedic implants that were removed for both presumed aseptic reasons and suspected infection (Ponraj et al., 2022). SF samples were further subjected to culture-dependent and culture-independent analyses. Thereby, an aNGS approach was employed to detect and phylotype *C. acnes*, and this along with the patients’ clinical information as well as whole-genome sequencing of the *C. acnes* isolates was used to differentiate *C. acnes* contamination from true infection.

The aim of this pilot study was to determine if mNGS of SF provides an additional value in the diagnosis of OIAIs due to *C. acnes*. Therefore, mNGS of SF from 24 implants was carried out, and the mNGS data were compared to other data available regarding these implants, including aNGS and tissue culture results. The results show that *C. acnes* was more often detected by mNGS than by culture and could potentially help to identify cases of *C. acnes* OIAIs that might be missed by culture alone.

## 2 Methods

### 2.1 Sample selection

In a previous study, 100 implants removed during revision surgery were collected between August 2019 and September 2020 and processed by sonication (Ponraj et al., 2022). SF specimens from 24 of the 100 implants were included in this pilot study. The selection was based on the SF culture results, and the included specimens were divided into three groups: group I, culture-negative (n = 4); group II, culture-positive for *C. acnes* (n = 10); and group III, culture-positive for other bacteria, i.e., staphylococci and *Finegoldia magna* (n = 10). In addition, normal saline that was sonicated and processed like SF from implants was included as the negative control.

### 2.2 DNA extraction from sonication fluid

Implants removed during revision surgeries were processed by the previously described vortex-sonication method (Borens et al., 2013). The resultant SF was split into two samples, one for culture-dependent analyses and another for culture-independent analyses. The samples for culture-independent analyses were stored at −20°C until processing. DNA extraction of the SF and the negative control was described previously (Ponraj et al., 2022). In brief, SF (40 ml) stored at −20°C was thawed overnight at 4°C, concentrated by centrifugation (15,000 × *g* for 1 h at 16°C using rotor F 13–14 × 50 Cy, Thermo Scientific™ Sorvall™ RC 6 Plus Centrifuge), and DNA extraction was performed using the DNeasy PowerSoil Kit (QIAGEN, Hilden, Germany) as per manufacturer’s instructions. DNA concentrations were measured using the Qubit dsDNA HS Assay (Thermo Fisher Scientific, Waltham, MA, USA) with a Qubit fluorometer. The same DNA extraction kit was used for the 24 samples to exclude any possible batch effect.

### 2.3 Library preparation and Illumina HiSeq sequencing

Illumina shotgun libraries were prepared using the Illumina DNA Prep Tagmentation Kit and Nextera DNA CD Indexes for multiplexing as recommended by the manufacturer (Illumina, San Diego, CA, USA). To assess the quality and size of the libraries, samples were run on an Agilent Bioanalyzer 2100 using an Agilent High Sensitivity DNA Kit as recommended by the manufacturer (Agilent Technologies, Waldbronn, Germany). The concentration of the libraries was determined using the Qubit dsDNA HS Assay Kit as recommended by the manufacturer (Life Technologies GmbH, Darmstadt, Germany). Sequencing was performed by using the NovaSeq6000 instrument (Illumina Inc., San Diego, CA, USA) using the NovaSeq6000 SP Reagent Kit (v. 1.5) and the NovaSeq XP 2-Lane Kit (v. 1.5) for sequencing in the paired-end mode 2 × 250 cycles.

### 2.4 Bioinformatics

The raw sequencing data were paired, and the paired-end reads along with metadata were uploaded and analysed using the publicly available web-based MG-RAST pipeline (https://www.mg-rast.org/) (Meyer et al., 2019). The workflow involves the following steps: 1) normalization or quality control where artificial duplicate reads are removed along with quality- and length-based trimming; 2) screening of sequences for potential protein-coding genes using a BLASTX search against multiple databases; 3) functional assignments and taxonomic distributions using the matches to external databases. To further analyse sequence reads that were assigned to *Cutibacterium* sp. or *Finegoldia* sp., the reads were extracted in MG-RAST and mapped to their corresponding genomes. Complete genomes of *C. acnes* KPA171202, *Cutibacterium avidum* 44067, *F. magna* ATCC29328, and “*Finegoldia nericia*” 09T494 were used as reference genomes and subsequently indexed using Bowtie2 (v. 2.5.0) (Langmead and Salzberg, 2012). The extracted query reads were mapped to the reference genomes using Bowtie2, and the resulting SAM-files were converted into BAM-files and indexed using Samtools (v. 1.6) (Li et al., 2009). Resulting BAM-files and reference genomes were imported into Geneious Prime (v. 2023.0.1) to visualize mapping results.

In an additional analysis, the mNGS data were analysed using Kraken2/Bracken. First, human DNA reads were removed as follows: paired-end reads were mapped against the Genome Reference Consortium human genome build 38 (GRCh38) using Bowtie2 (v. 2.5.1) (Langmead and Salzberg, 2012). Any reads concordantly or discordantly mapped to the human reference genome were removed from the samples using Samtools (v. 1.6) and Bedtools (v. 2.30.0). The resulting filtered FASTQ-files were classified using Kraken2 (v. 2.1.2) (Wood et al., 2019) using a database consisting of bacteria, archaea, viruses, and plasmids with a minimum hit group of 3. The classification was subsequently re-estimated using Bracken (v. 2.5.0) (Lu et al., 2017). Since the Kraken2/Bracken analysis is more sensitive, resulting in more taxonomically assigned reads than MG-RAST, we introduced a minimum read count threshold of 150 (corresponding to approx. 0.1% of the total amount of bacterial reads per sample). Four samples had *C. acnes* reads <150, i.e., samples 2 and 3 in group II, and samples 9 and 10 in group III.

### 2.5 Amplicon NGS control PCR

To test the sensitivity of the aNGS approach (in detail described previously in Ponraj et al., 2022), a PCR was performed with the single-locus sequence typing (SLST) primers (5′-TTGCTCGCAACTGCAAGCA-3′ and 5′-CCGGCTGGCAAATGAGGCAT-3′) and different amounts (1,000 to 0.001 pg) of *C. acnes* genomic DNA (strain 266). The PCR contained 5 μl of genomic DNA template, 2.5 μl of AccuPrime PCR Buffer II (Invitrogen, Waltham, MA, USA), 1.5 μl of each primer (10 μM; DNA Technology, Risskov, Denmark), 0.15 μl of AccuPrime Taq DNA Polymerase High Fidelity (Invitrogen, Waltham, MA, USA), and 14.35 μl of PCR-grade water. The PCR was performed using the following cycle conditions: initial denaturation at 94°C for 2 min, 35 cycles of denaturation at 94°C for 20 s, annealing at 55°C for 30 s, elongation at 68°C for 1 min, and final elongation step at 72°C for 5 min.

### 2.6 Ethical approval

The study was registered with Region Midtjylland with reference number 661624. The Central Denmark Region ethical committee waived the need for ethical approval.

### 2.7 Statistical analyses

Data were entered in Excel, and graphs and charts were prepared using Prism GraphPad.

## 3 Results

**Figure 1:**
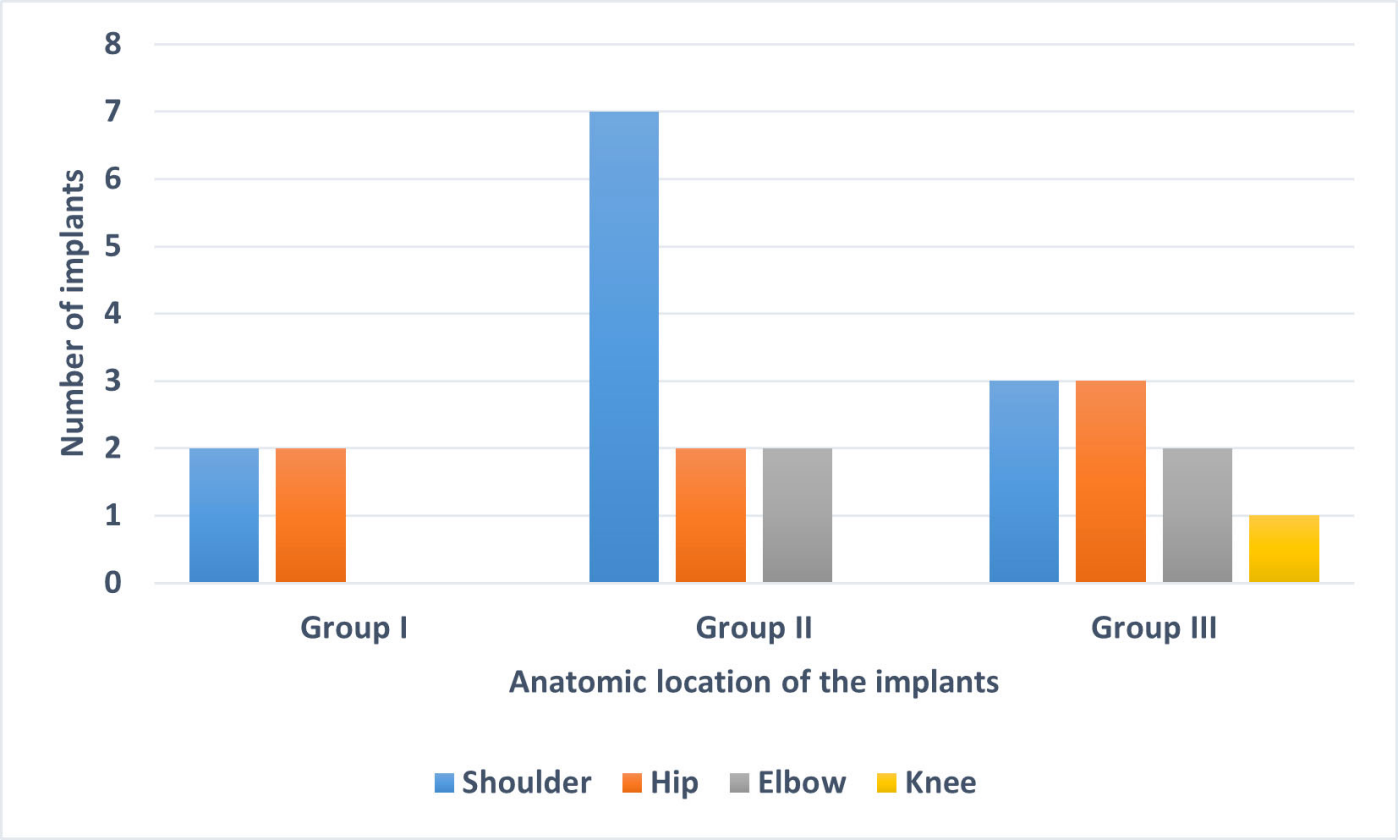
Anatomic location of the 24 implants used in this study. The implants were divided into three groups based on sonication fluid culture results: Group I: culture-negative, Group II: culture-positive for *C. acnes* and Group III: culture-positive for organisms other than *C. acnes*.

Sequencing (mNGS) of SF specimens from 24 implants, divided into three groups (group I, culture-negative (n = 4); group II, culture-positive for *C. acnes* (n = 10); group III, culture-positive for other bacteria (n = 10)) was performed. The anatomical locations of the selected 24 implants from the three groups are shown in **Figure 1**. The majority of the implants were from the shoulder, followed by the hip.

Shotgun sequencing of DNA extracted from the 24 SF samples resulted in an average of 8,437,406 sequence reads per sample (range, 5,898,825–12,907,838). The data were first analysed with MG-RAST for taxonomic assignment of sequence reads. The total numbers of sequence reads, and sequence reads that passed quality control are given in **Table S1**. The average number of reads that passed quality control was 778,496 (range, 443,276–1,424,198) (9.2%). The top 10 genera assigned by MG-RAST for the 24 SF samples as well as the number of reads and percentage of total reads for each genus are given in **Table S2**. No sequence reads were obtained from the saline control sample.

**Figure 2:**
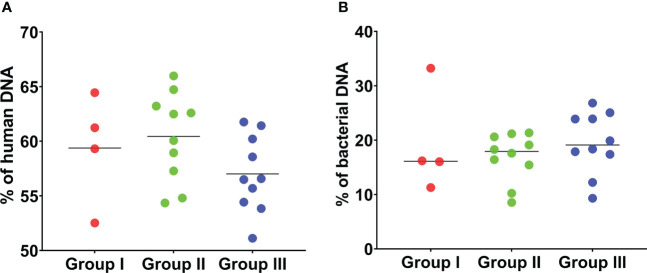
Percentage of human and bacterial DNA detected by mNGS of 24 sonication fluid samples. **(A)** The percentage of human DNA detected by mNGS in the 24 SF samples in the three groups is shown. The percentage of human DNA is similar across the three groups. **(B)** The percentage of bacterial DNA detected by mNGS is shown. The percentage of bacterial DNA across the three groups is much lower than the percentage of human DNA in the three groups. This analysis is based on MG-RAST assignment results.

Overall, the percentage of human DNA across the 24 SF samples in the three groups was higher than the percentage of bacterial DNA (**Figures 2A, B**). Reads that matched non-bacterial genera like *Canis*, *Macaca*, *Drosophila*, and *Danio* were also detected in all SF samples (**Table S2**). In addition, several SF samples showed the apparent presence of DNA from gut bacteria like the genera *Coprobacillus* (n = 23), *Bacteroides* (n = 13), *Prevotella* (n = 10), and *Clostridium* (n = 7). We also used an alternative approach to analyse the data, by first filtering and discarding reads that matched the human genome and subsequently analysing the remaining reads with Kraken2/Bracken for taxonomic assignment (**Table 1**).

**Table 1:** Comparison of SF culture results with results of mNGS and aNGS of SF of the 24 implants included in this pilot study.

S. no.	Reason for implant removal*	Tissue culture	SF culture		aNGS of SF	mNGS of SF		
			Microorganism	CFU/ml **		Microorganism	MG-RAST: reads (% of total bacteria)	Kraken2: reads (% of total bacteria)
Group I—SF culture-negative								
1	Pain from plate/screw	n.g.	n.g.	–	Neg.	*Cutibacterium acnes*	–	–
2	Aseptic loosening	CoNS (1/5) *C. acnes* (1/5)	n.g.	–	Neg.	*C. acnes*	–	–
3	Unknown	n.d.	n.g.	–	Neg.	*C. acnes*	–	–
4	Suspected OIAIs	n.g.	n.g.	–	n.d.	*C. acnes*	793 (8.4)	1,240 (1)
Group II—Culture-positive for *C. acnes*								
1	Aseptic failure	n.g.	*C. acnes*	(20)	Neg.	*C. acnes*	–	–
2	Pain from plate	n.d.	*C. acnes*	170	Pos.	*C. acnes*	–	–
3	Aseptic loosening	*C. acnes* (4/5)	*C. acnes*	100	Pos.	*C. acnes*	–	–
4	Aseptic loosening	*C. acnes* (2/5) *Staphylococcus epidermidis* (2/5)	*C. acnes*	>250	Pos.	*C. acnes*	463 (2.5)	821 (0.8)
5	Aseptic failure	*C. acnes* (2/5)	*C. acnes*	60	Neg.	*C. acnes*	545 (3.0)	961 (1)
6	Pain from plate	n.d.	*C. acnes*	90	Pos.	*C. acnes*	488 (4.2)	890 (0.4)
7	Suspected OIAIs	n.g.	*C. acnes*	(10)	Pos.	*C. acnes*	2,918 (13.1)	5,113 (4)
8	Aseptic failure	*C. acnes* (5/5)	*C. acnes*	(20)	Pos.	*C. acnes*	2,204 (12.0)	4,101 (4)
9	Suspected OIAIs	*C. acnes* (4/5)	*C. acnes*	100	Pos.	*C. acnes*	401 (3.2)	652 (1)
10	Pain from plate	n.d.	*C. acnes*	>250	Pos.	*C. acnes*	1,883 (20.5)	3,257 (3)
Group III—Culture-positive for other bacteria								
1	Suspected OIAIs	*S. epidermidis* (5/5)	*S. epidermidis*	>250	Neg.	*S. epidermidis*	395 (1.9)	574 (0.8)
2	Aseptic failure	n.g.	*S. epidermidis*	(20)	n.d.	*S. epidermidis*	–	–
3	Glenoid attrition	n.g.	*S. epidermidis*	(30)	n.d.	*S. epidermidis*	–	–
4	Suspected OIAIs	*Staphylococcus aureus* (4/5)	*S. aureus*	>250	Neg.	*S. aureus* *C. acnes*	5,322 (29.5) 395 (2.2)	11,620 (11) 583 (0.6)
5	Aseptic failure	n.g.	*Cutibacterium avidum*	(200)***	n.d.	*C. acnes*	3,140 (14.2)	5,932 (4)
6	Suspected OIAIs	*Finegoldia magna* (2/5)	*F. magna*	>250	n.d.	*F. magna* *C. acnes*	9,184 (41.7) 564 (2.6)	15,340 (17) 923 (1)
7	Suspected OIAIs	*S. aureus* (3/5)	*S. aureus*	>250	Neg.	*S. aureus* *C. acnes*	1,208 (5.2) 2,449 (10.6)	5,544 (4) 3,760 (3)
8	Pain from plate	n.d.	*S. aureus*	>250	Neg.	*S. aureus* *C. acnes*	217 (2.5) 458 (5.3)	2,862 (4) 723 (1)
9	Suspected OIAIs	*S. aureus* (5/5)	*S. aureus*	>250	Neg.	*S. aureus*	5,932 (40.3)	12,221 (14)
10	Fracture	n.g.	*S. epidermidis*	>250	n.d.	*S. epidermidis*	187 (3.0)	347 (0.5)

n.g., no growth; n.d., not determined; CoNS, coagulase-negative Staphylococcus; SF, sonication fluid; mNGS, metagenomic next-generation sequencing; aNGS, amplicon-based next-generation sequencing; CFU, colony-forming unit; OIAIs, orthopaedic implant-associated infections; EBJIS, European Bone and Joint Infection Society.

*Based on pre-operative diagnosis of the operating surgeon.

**CFU counts below the cutoff described in the latest EBJIS criteria (>50 CFU/ml for uncentrifuged SF; >200 CFU/ml for centrifuged SF) are in brackets.

***Centrifuged SF.

In the following, mNGS results are outlined for the three groups and presented in the context of other results, i.e., SF culture and aNGS data regarding the detection of *C. acnes*.

- Group I—culture negative: in three out of four SF specimens in group I, no sequence reads matching *C. acnes* were detected by mNGS. This corresponded with aNGS results, as all three samples were negative for *C. acnes* by SLST PCR (Ponraj et al., 2022). In one SF sample (group I #4), *C. acnes* DNA was detected (793 reads (MG-RAST); 1,240 reads (Kraken2/Bracken)) (**Table 1**). The corresponding implant was both SF and tissue culture-negative; aNGS was not performed for this SF specimen. The implant was removed due to suspected infection diagnosed by the presence of a sinus tract, so *C. acnes* could potentially represent the causative agent that was missed by culture.
- Group II—culture-positive for *C. acnes*: group II included 10 samples that were SF culture-positive for *C. acnes* (**Table 1**). mNGS detected sequence reads matching *C. acnes* in seven of the 10 samples in this group. In these seven samples, 401 to 2,918 *C. acnes* sequence reads (average 1,271 reads) were identified with MG-RAST, corresponding to 2.5% to 20.5% of all detected bacterial reads. A slightly higher number of *C. acnes* reads were found with the Kraken2/Bracken approach (average 2,256 reads). Regarding the three samples, for which no *C. acnes* reads were obtained, one of them (group II #1) was also aNGS-negative (**Table 1**). In addition, the SF culture showed *C. acnes* growth with a low colony-forming unit (CFU) count of only 20 CFU/ml, and the five tissue cultures from that implant had no bacterial growth. Thus, *C. acnes* in this case could likely be a contaminant, possibly obtained at the cultivation step. The second sample (group II #2) with no *Cutibacterium* sp. reads was aNGS-positive. However, tissue culture results were not available for this sample (**Table 1**). The third sample (group II #3) was tissue culture-positive for *C. acnes*. This sample had a low *C. acnes* read count (49 and 81 reads assigned by MG-RAST and Kraken2/Bracken, respectively), which was under the applied read count threshold.
- Group III—culture-positive for other bacteria: 10 SF samples that were SF culture-positive for bacteria other than *C. acnes* were included in group III. The bacterial species identified from SF culture (*Staphylococcus epidermidis*, *Staphylococcus aureus*, *F. magna*, and *C. avidum*) and their corresponding tissue culture results are listed in **Table 1**. In eight out of 10 samples in this group, the bacterial genus determined by mNGS matched the bacterial genus detected by SF culture (**Table 1**). The two samples (group III #2 and #3) with discordant results had both growth of *S. epidermidis* on SF culture, albeit with a low CFU count of 20–30 CFU/ml. Interestingly, in four samples (group III #4, #6, #7, and #8), in addition to the bacteria detected by SF culture (3 × *S. aureus*; 1 × *F. magna*), sequence reads from *C. acnes* (395 to 2,449 reads (MG-RAST); 2.2% to 10.6% of bacterial reads) were detected. In two of these samples (group III #7 and #8), there were twice as many *C. acnes* reads detected than staphylococcal reads, according to the MG-RAST analysis (**Table 1**). Three of the samples (group III #4, #7, and #8) were negative by aNGS, while aNGS was not performed in the remaining sample (group III #6). This raised the question of how sensitive the aNGS approach is. We therefore tested the aNGS PCR with different amounts of genomic DNA of *C. acnes*. The PCR detection limit was determined to be a minimum amount of 0.1 pg *C. acnes* template DNA, which corresponded to ca. 37 genome copies (**Supplementary Figure 1**).

**Figure 3:**
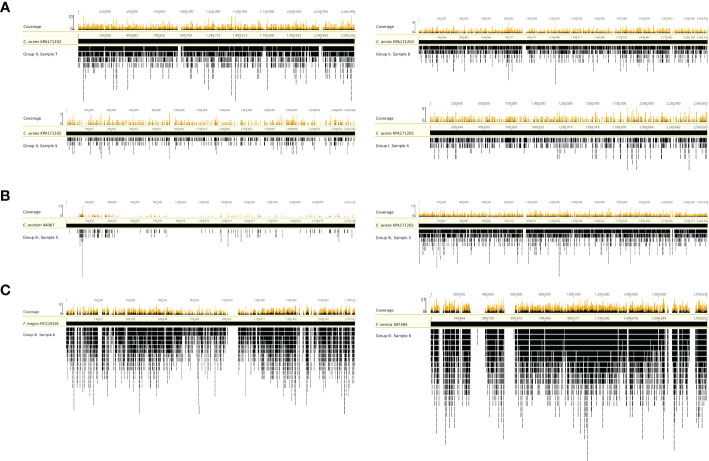
Mapping of sequence reads assigned to *Cutibacterium* sp. and/or *Finegoldia* sp. to their corresponding reference genomes. **(A)** Mapping of sequence reads assigned to *Cutibacterium* sp. from four samples (sample 4 from group I, samples 5,7 and 8 from group II) to the reference genome of *C. acnes* KPA171202. **(B)** Mapping of sequence reads assigned to *Cutibacterium* sp. from sample 5 in group III to the genome of *C. avidum* 44067 and to the genome of *C. acnes* KPA171202. **(C)** Mapping of sequence reads corresponding to *Finegoldia* sp. from sample 6 in group III to the genome of *Finegoldia magna* ATCC29328 and “*Finegoldia nericia*” 09T494. This analysis is based on MG-RAST assignment results.

To test whether the obtained sequence reads by mNGS were originating from the entire bacterial genome or were potential artefacts, we mapped extracted sequence reads to reference genomes. Four samples (group I #4 and group II #5, #7, and #8) with *C. acnes* reads were selected and mapped against the *C. acnes* reference genome of strain KPA171202 (**Figure 3A**). The results showed that the sequence reads obtained by mNGS originated from the entire *C. acnes* genome and not only from certain conserved parts, such as rRNA genes. In one sample (group III #5), whose SF was culture-positive for *C. avidum*, mNGS detected 3,140 and 5,932 sequence reads assigned to *C. acnes* based on MG-RAST and Kraken2/Bracken analysis, respectively. To test if these reads originated from *C. acnes* or *C. avidum*, as culture results suggested, sequence reads were mapped against the genomes of *C. avidum* 44067 and *C. acnes* KPA171202 (**Figure 3B**). Results indicated that most reads originated from *C. acnes* and not from *C. avidum*. In another sample (group III #6), *F. magna* was identified by culture. *F. magna* is closely related to the tentative species “*F. nericia*” (Brüggemann et al., 2018). The 9,184 sequence reads assigned to *Finegoldia* sp. by MG-RAST were mapped against the genomes of *F. magna* ATCC29328 and “*F. nericia*” 09T494 (**Figure 3C**). Results showed that the detected DNA originated mainly, if not entirely, from “*F. nericia*”.

## 4 Discussion

The significance of *C. acnes* isolated from removed orthopaedic implants and their surrounding tissues, including the possibility of overdiagnosis due to the interpretation of contamination or commensal colonization as infection, has often been debated (Hudek et al., 2014; Patel et al., 2020; Falstie-Jensen et al., 2021; Hudek et al., 2021). At the same time, the risk of underdiagnosing *C. acnes* OIAIs because of the slow-growing nature of the bacterium has also been discussed (Butler-Wu et al., 2011; Aubin et al., 2014; Bossard et al., 2016).

In this pilot study, a shotgun sequencing approach of SF was used that could potentially help to identify cases of *C. acnes* OIAIs that were not detected by culture. A total of 24 SF samples, based on their SF culture results, were included and divided into three groups. A large number of microbial sequence reads were obtained in all SF samples including the SF culture-negative samples. As pointed out in several recent studies, particular care needs to be taken when applying mNGS to low-biomass samples, as low-biomass samples are prone to contamination derived from DNA that could be present in liquids or reagents used, such as DNA extraction kits or reagents used for NGS library preparations (Weiss et al., 2014; Street et al., 2017; Thoendel et al., 2017; Thoendel et al., 2018; Eisenhofer et al., 2019). In our study, no DNA was detected by mNGS when a mock sample was sequenced, i.e., the saline used for sonication.

In all SF specimens, sequence reads that originated from gut bacteria were detected by mNGS (**Table S2**). The origin of these sequence reads in SF samples remains to be investigated. DNA of the gut microbiome in SF could potentially be due to gut permeability, which has been linked to OIAIs *via* the “gut–immune–joint axis” (Chisari et al., 2022), but this does not explain the presence of gut microbial DNA in culture-negative samples with no clinical indication of infection. Another possibility is that DNA of gut bacteria could be present in the normal joint microenvironment, as some studies suggested the presence of bacteria or DNA thereof in native joints (Torchia et al., 2020; Clarkson et al., 2022).

Regarding *C. acnes* in particular, several previous studies suggested that *C. acnes* or rather its DNA is a common contaminant in mNGS data, where it is assumed that *C. acnes* DNA is present in reagents needed for DNA isolation (e.g., DNA extraction kits) and sequence library preparation (Mollerup et al., 2016; Street et al., 2017). However, we did not detect *C. acnes* DNA in the control sample (saline). Moreover, *C. acnes* DNA was only detected in 14/24 samples and not in all samples, as would be expected if *C. acnes* DNA is a common contaminant. This speaks against a general contamination issue with *C. acnes* DNA in the used saline solution or DNA extraction kit or sequencing reagents. However, it cannot be excluded that *C. acnes* could have contaminated the implant in the process of surgery/implant removal in some but not all cases. Such surgery-related contamination is very difficult to prove or disprove. It has previously been reported that carriage of *C. acnes* on human skin can vary substantially from patient to patient and from body site to body site, which can lead to *C. acnes* wound contamination in some but not all patients (McLorinan et al., 2005; Patrick et al., 2017; Patel et al., 2020; Shroff et al., 2023). For example, the shoulder site is usually more heavily colonized with *C. acnes*, especially in male patients (Patel et al., 2020; Hudek et al., 2021; Shroff et al., 2023). A potential possibility to reduce wound contamination could be to wash the removed implants before sonication; this was not performed in this study due to logistic reasons. Here, the implants were placed in sterile, airtight, single-use plastic containers in the operating room and covered with sterile saline before being transported to the lab (Ponraj et al., 2022).

In the culture-negative group, sequence reads from *C. acnes* were obtained in only one sample that was both SF and tissue culture-negative. Clinically, the respective implant was defined as infected due to the presence of a sinus tract. Thus, it could potentially represent a *C. acnes* infection that was missed by culture. Alternatively, *C. acnes* or its DNA contaminated the sample at the DNA extraction step or later. Both explanations are possible, as the risk of contamination in low-biomass mNGS studies (as outlined above) as well as the ability of mNGS to identify organisms not detected by culture has been described previously in multiple studies (Street et al., 2017; Ivy et al., 2018; Sanderson et al., 2018; Weaver et al., 2019; Cai et al., 2020; Wang et al., 2020; He et al., 2021; Noone et al., 2021; Street et al., 2022; Tan et al., 2022).

Of the 10 samples in group II that had growth of *C. acnes* in SF culture, reads from *C. acnes* were obtained in seven samples. *C. acnes* isolates from two of the remaining three samples (#1 and #2) had been classified as likely contamination in our previous study (Ponraj et al., 2022), in which a combined analysis of genome sequencing and SLST typing of *C. acnes* isolates, aNGS of SF, tissue culture results, and pre-operative clinical diagnosis was used to determine the significance of *C. acnes* detection.

In four samples from group III that included SF culture-positive samples for bacteria other than *C. acnes*, mNGS detected *C. acnes* reads in addition to the reads from the bacteria also detected by SF culture, i.e., *S. aureus* in three cases and *F. magna* in one case. This could either represent *C. acnes* DNA contamination during mNGS or be a polymicrobial infection that was not detected in culture because the slow-growing *C. acnes* was overgrown or inhibited by the other faster-growing organism, i.e., *S. aureus* and *F. magna*. Interestingly, in two samples, there were twice as many *Cutibacterium* sp. reads detected than *S. aureus* reads (using the MG-RAST pipeline), albeit only *S. aureus* was identified by SF culture. This suggests that a polymicrobial infection could be present, which was missed by culture since *S. aureus* grows rapidly and possibly inhibits the growth of *C. acnes*.

It is challenging to determine and compare the detection limit of the two methods, mNGS and aNGS. Here, we determined in a mock setting that the aNGS method has a detection limit of ca. 37 *C. acnes* genome copies. However, in real samples, such as SF specimens, which potentially contain much DNA of human origin that could interfere with the *C. acnes* SLST PCR, the number of *C. acnes* genome copies needed to obtain a positive PCR result will likely be higher. Regarding the mNGS method, the detection limit has not been experimentally determined so far; it will depend on many internal and external factors, such as the sequencing specificities (platform, materials, sequencing depths, and bioinformatics pipeline) as well as the relative abundance of *C. acnes* DNA in a given sample. Our data here suggested that mNGS is more sensitive than aNGS since four out of 10 aNGS-negative samples had actually *C. acnes* reads above the read count threshold. Like in other mNGS projects, a read count threshold was set (150 reads, corresponding to approx. 0.1% of the total amount of bacterial reads per sample) to eliminate potential false positives (Street et al., 2017). Some samples had read counts below the threshold, e.g., MG-RAST and Kraken2/Bracken detected one and four samples with low *C. acnes* read count (on average 51 reads) and four and 13 samples with low *S. epidermidis* read count (on average 37 reads). This might represent NGS-derived contamination, as low counts of *S. epidermidis* and *C. acnes* reads were previously reported in negative controls used in shotgun sequencing projects (Sanabria et al., 2020).

This pilot study has several limitations. The number of samples is limited due to the exploratory nature of the study. Optimal read count thresholds to filter out potential contaminant reads were not established in the study due to the lack of mock community mNGS data (with defined *C. acnes* genome copy numbers) and also hampered by the lack of detecting bacterial reads in negative control samples. Tissue sample culture and aNGS were not performed for all samples included in the study, and finally, due to lack of clinical follow-up, the significance of *C. acnes* reads detected by mNGS, especially in groups I and III, could not be determined. Further studies with the inclusion of clinical follow-up are needed before mNGS can be recommended for the diagnosis of OIAIs due to *C. acnes*.

## 5 Conclusions

The current study shows that *C. acnes* DNA was detected in more samples by mNGS compared to the culture of SF. Additional identification of *C. acnes* was seen in one culture-negative SF sample and in four SF samples that were culture-positive for other bacteria. Thus, mNGS could possibly be used to identify cases of potential *C. acnes* OIAIs, especially in polymicrobial infections that might be overlooked or misinterpreted by culture-based detection alone.

## Data availability statement

The datasets presented in this study can be found in online repositories. The names of the repository/repositories and accession number(s) can be found below: SRA with the accession number PRJNA940664.

## Ethics statement

The studies involving human participants were reviewed and approved by Ethical Committee Region Midtjylland, Denmark. The patients/participants provided their written informed consent to participate in this study.

## Author contributions

DP, JL, TF-J, NJ, CR, and HB contributed to the conception and design of the study. DP performed wet lab benchwork. DP analysed the data. ML, AP, AH, and HB contributed to sequence data generation and data analyses. DP and HB wrote the manuscript. All authors contributed to the article and approved the submitted version.

## Supplementary material

Supplementary Figure 1 SLST-PCR with different concentrations of *C. acnes* genomic DNA as template. *C. acnes* strain 266 was used. The lanes: 1, DNA ladder (EasyLadder I); 2, 1000 pg; 3, 100 pg; 4, 10 pg; 5, 1 pg; 6, 0.1 pg; 7, 0.01 pg; 8, 0.001 pg; 9, negative control; 10, positive control (1 ng *C. acnes* DNA).
