# Supplementary figures and images for: Shotgun sequencing of sonication fluid for the diagnosis of orthopaedic implant-associated infections with Cutibacterium acnes as suspected causative agent
Source: Front Cell Infect Microbiol. 2023 May 17;13:1165017. doi: 10.3389/fcimb.2023.1165017 (PMC10229904; doi:10.3389/fcimb.2023.1165017)

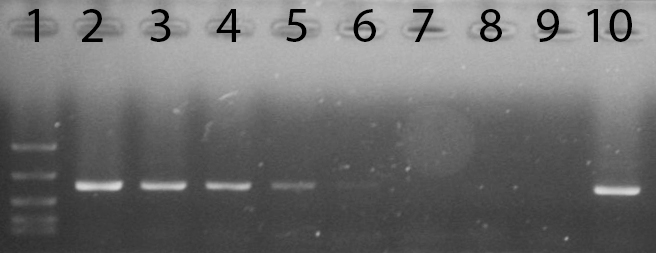

Supplement: Supplementary Figure 1 — SLST-PCR with different concentrations of C. acnes genomic DNA as template. C. acnes strain 266 was used. The lanes: 1, DNA ladder (EasyLadder I); 2, 1000 pg; 3, 100 pg; 4, 10 pg; 5, 1 pg; 6, 0.1 pg; 7, 0.01 pg; 8, 0.001 pg; 9, negative control; 10, positive control (1 ng C. acnes DNA). [file Image_1.jpeg]
