# Supplementary material for: Shotgun sequencing of sonication fluid for the diagnosis of orthopaedic implant-associated infections with Cutibacterium acnes as suspected causative agent
Source: Front Cell Infect Microbiol. 2023 May 17;13:1165017. doi: 10.3389/fcimb.2023.1165017 (PMC10229904; doi:10.3389/fcimb.2023.1165017)
Supplement: Supplementary file 2 [file Table_1.docx]

**Supplementary Table S1: mNGS data of SF specimens of 24 implants: total number of sequence reads, and sequence reads that passed quality control/filter using the MG-RAST and Kraken 2/Braken pipelines**

| **S. No** | **Sequence file name** | **Total sequence reads (MG-RAST)** | **Sequence reads that passed quality control**  **(MG-RAST)** | **Sequence reads that passed filter**  **(Kraken 2)** |
| --- | --- | --- | --- | --- |
| **Group I** |  |  |  |  |
| **1** | HBRU_m_7 | 9,162,830 | 756,492 | 115,778 |
| **2** | HBRU_m_8 | 8,217,831 | 476,755 | 77,683 |
| **3** | HBRU_m_16 | 9,521,326 | 745,847 | 94,572 |
| **4** | HBRU_m_26 | 8,073,326 | 985,074 | 89,600 |
| **Group II** |  |  |  |  |
| **1** | HBRU_m_11 | 10,154,892 | 521,853 | 89,031 |
| **2** | HBRU_m_13 | 9,115,032 | 657,219 | 90,177 |
| **3** | HBRU_m_14 | 10,006,201 | 871,966 | 120,256 |
| **4** | HBRU_m_15 | 9,070,914 | 852080 | 107,105 |
| **5** | HBRU_m_22 | 7,456,760 | 791,777 | 98,828 |
| **6** | HBRU_m_24 | 12,907,838 | 1,424,198 | 199,849 |
| **7** | HBRU_m_27 | 8,607,564 | 1,037,936 | 135,614 |
| **8** | HBRU_m_28 | 7,117,117 | 872,496 | 95,168 |
| **9** | HBRU_m_29 | 5,898,825 | 564,546 | 67,339 |
| **10** | HBRU_m_31 | 8,066,020 | 897,200 | 104,782 |
| **Group III** |  |  |  |  |
| **1** | HBRU_m_9 | 8,182,060 | 449,689 | 72,518 |
| **2** | HBRU_m_10 | 8,318,549 | 458,038 | 77,090 |
| **3** | HBRU_m_12 | 8,496,536 | 443,276 | 70,391 |
| **4** | HBRU_m_18 | 8,821,411 | 1,001,706 | 103,087 |
| **5** | HBRU_m_19 | 8,997,090 | 1,129,967 | 134,084 |
| **6** | HBRU_m_20 | 6,118,355 | 610,244 | 88,882 |
| **7** | HBRU_m_21 | 9,147,157 | 1,004,754 | 129,658 |
| **8** | HBRU_m_23 | 6,572,294 | 662,755 | 68,552 |
| **9** | HBRU_m_25 | 7,233,341 | 767,898 | 87,941 |
| **10** | HBRU_m_30 | 7,234,466 | 700,128 | 76,967 |
