# Supplementary material for: Shotgun sequencing of sonication fluid for the diagnosis of orthopaedic implant-associated infections with Cutibacterium acnes as suspected causative agent
Source: Front Cell Infect Microbiol. 2023 May 17;13:1165017. doi: 10.3389/fcimb.2023.1165017 (PMC10229904; doi:10.3389/fcimb.2023.1165017)
Supplement: Supplementary file 3 [file Table_2.docx]

**Supplementary Table S2: mNGS data analysis with MG-RAST: top ten genera in the 24 SF samples across the three groups with number of reads in each genus and percentage of reads given in parentheses**

| **S. No** | **Top ten genera – number of reads (percentage)** |
| --- | --- |
| **Group I** | |
| **1** | *Homo* - 40,019 (64.45%), *Macaca* - 4,928 (7.94%), *Candidatus phytoplasma* - 3,847 (6.20%), *Canis* - 2,433 (3.92%), *Coprobacillus* - 1,978 (3.19%), *Drosophila* - 976 (1.57%), *Bacteroides* - 951 (1.53%), *Cyanothece* - 770 (1.24%), *Prevotella* - 718 (1.16%), Pan - 595 (0.96%) |
| **2** | *Homo* - 30,274 (52.52%), *Macaca* - 3,542 (6.14%), *Canis* - 3,071 (5.33%), *Bacteroides* - 2,796 (4.85%), *Candidatus phytoplasma* - 2,680 (4.65%), *Prevotella* - 2,210 (3.83%), *Coprobacillus* - 1,907 (3.31%), *Drosophila* - 668 (1.16%), *Clostridium* - 622 (1.08%), *Cyanothece* - 537 (0.93%) |
| **3** | *Homo* - 36,411 (61.24%), *Macaca* - 5,325 (8.96%), *Candidatus phytoplasma* - 3,398 (5.71%), *Canis* - 3,377 (5.68%), *Coprobacillus* - 2,460 (4.14%), *Bacteroides* - 966 (1.62%), *Drosophila* - 951 (1.60%), *Cyanothece* - 709 (1.19%), *Prevotella* - 612 (1.03%), Pan - 568 (0.96%) |
| **4** | *Homo* - 28,975 (59.31%), *Macaca* - 6,058 (12.40%), *Candidatus phytoplasma* - 2,851 (5.84%), *Coprobacillus* - 1,874 (3.84%), *Canis* - 1,663 (3.40%), *Cyanothece* - 811 (1.66%), *Drosophila* - 806 (1.65%), *Propionibacterium* - 793 (1.62%), Pan - 782 (1.60%), *Alkaliphilus* - 614 (1.26%) |
| **Group II** | |
| **1** | *Homo* - 41,811 (64.73%), *Canis* - 4,597 (7.12%), *Macaca* - 4,203 (6.51%), *Candidatus phytoplasma* - 2,596 (4.02%), *Drosophila* - 1,816 (2.81%), *Bacteroides* - 1,088 (1.68%), *Prevotella* - 1,014 (1.57%), *Cyanothece* - 831 (1.29%), *Danio* - 525 (0.81%), Pan - 444 (0.69%) |
| **2** | *Homo* - 37,170 (63.22%), *Macaca* - 4,778 (8.13%), *Canis* - 3,407 (5.79%), *Candidatus phytoplasma* - 3,217 (5.47%), *Coprobacillus* - 2,267 (3.86%), *Bacteroides* - 978 (1.66%), *Prevotella* - 755 (1.28%), *Drosophila* - 634 (1.08%), *Cyanothece* - 615 (1.05%), Pan - 528 (0.90%) |
| **3** | *Homo* - 41,832 (60.06%), *Macaca* - 5,370 (7.71%), *Canis* - 4,050 (5.81%), *Candidatus phytoplasma* - 3,371 (4.84%), *Coprobacillus* - 2,290 (3.29%), *Bacteroides* - 1,368 (1.96%), *Prevotella* - 1,277 (1.83%), *Drosophila* - 845 (1.21%), *Cyanothece* - 805 (1.16%), Pan - 657 (0.94%) |
| **4** | *Homo* - 32,503 (57.28%), *Macaca* - 5,361 (9.45%), *Candidatus phytoplasma* - 3,037 (5.35%), *Canis* - 2,937 (5.18%), *Coprobacillus* - 2,084 (3.67%), *Bacteroides* - 1,009 (1.78%), *Drosophila* - 915 (1.61%), *Prevotella* - 849 (1.50%), *Cyanothece* - 609 (1.07%), Pan - 576 (1.02%) |
| **5** | *Homo* - 36,000 (58.94%), *Macaca* - 5,450 (8.92%), *Vibrio* - 4,901 (8.02%), *Candidatus phytoplasma* - 2,515 (4.12%), *Coprobacillus* - 1,678 (2.75%), *Canis* - 1,482 (2.43%), *Thermosinus* - 1,120 (1.83%), *Drosophila* - 771 (1.26%), *Cyanothece* - 725 (1.19%), Pan - 642 (1.05%), *Propionibacterium* - 545 (0.89%) |
| **6** | *Homo* - 55,817 (65.98%), *Macaca* - 8,176 (9.66%), *Canis* - 4,494 (5.31%), *Candidatus phytoplasma* - 3,710 (4.39%), *Coprobacillus* - 2,636 (3.12%), *Cyanothece* - 1,031 (1.22%), Pan - 1,016 (1.20%), *Drosophila* - 767 (0.91%), *Thermosinus* - 712 (0.84%), *Danio* - 654 (0.77%) |
| **7** | *Homo* - 35,527 (54.80%), *Macaca* - 5,833 (9.00%), *Candidatus phytoplasma* - 2,958 (4.56%), *Propionibacterium* - 2,918 (4.50%), *Canis* - 2,439 (3.76%), *Bacteroides* - 2,277 (3.51%), *Aeromonas* - 2,202 (3.40%), *Coprobacillus* - 1,945 (3.00%), *Clostridium* - 1,561 (2.41%), *Drosophila* - 849 (1.31%) |
| **8** | *Homo* - 28,502 (54.35%), *Macaca* - 4,889 (9.32%), *Candidatus phytoplasma* - 2,745 (5.23%), *Propionibacterium* - 2,204 (4.20%), *Coprobacillus* - 1,906 (3.63%), *Canis* - 1,809 (3.45%), *Thermosinus* - 1,078 (2.06%), *Bacteroides* - 1,003 (1.91%), *Clostridium* - 998 (1.90%), *Aeromonas* - 957 (1.82%) |
| **9** | *Homo* - 27,105 (62.49%), *Macaca* - 3,759 (8.67%), *Candidatus phytoplasma* - 2,394 (5.52%), *Canis* - 1,594 (3.67%), *Coprobacillus* - 1,319 (3.04%), *Thermosinus* - 743 (1.71%), *Drosophila* - 607 (1.40%), *Clostridium* - 574 (1.32%), *Cyanothece* - 485 (1.12%), Pan - 447 (1.03%) |
| **10** | *Homo* - 35,159 (62.59%), *Macaca* - 5,495 (9.78%), *Canis* - 3,165 (5.63%), *Candidatus phytoplasma* - 2,994 (5.33%), *Coprobacillus* - 2,139 (3.81%), *Propionibacterium* - 1,883 (3.35%), *Cyanothece* - 783 (1.39%), Pan - 715 (1.27%), *Drosophila* - 714 (1.27%), *Pongo* - 242 (0.43%) |
| **Group III** | |
| **1** | *Homo* - 29,579 (56.57%), *Canis* - 3,814 (7.29%), *Macaca* - 3,302 (6.32%), *Candidatus phytoplasma* - 2,464 (4.71%), *Coprobacillus* - 2,032 (3.89%), *Bacteroides* - 1,627 (3.11%), *Prevotella* - 1,134 (2.17%), *Cyanothece* - 524 (1.00%), *Drosophila* - 523 (1.00%), *Staphylococcus* - 395 (0.76%) |
| **2** | *Homo* - 31,507 (58.57%), *Macaca* - 3,312 (6.16%), *Canis* - 2,679 (4.98%), *Candidatus phytoplasma* - 2,437 (4.53%), *Bacteroides* - 2,064 (3.84%), *Coprobacillus* - 1,566 (2.91%), *Prevotella* - 1,432 (2.66%), *Drosophila* - 553 (1.03%), *Cyanothece* - 492 (0.91%), *Clostridium* - 461 (0.86% |
| **3** | *Homo* - 33,893 (61.76%), *Macaca* - 3,638 (6.63%), *Canis* - 3,485 (6.35%), *Candidatus phytoplasma* - 3,438 (6.26%), *Coprobacillus* - 2,025 (3.69%), *Bacteroides* - 1,046 (1.91%), *Prevotella* - 956 (1.74%), *Drosophila* - 738 (1.34%), *Cyanothece* - 623 (1.14%), Pan - 412 (0.75%) |
| **4** | *Homo* - 34,223 (53.84%), *Macaca* - 6,173 (9.71%), *Staphylococcus* - 5,322 (8.37%), *Candidatus phytoplasma* - 3,453 (5.43%), *Canis* - 2,482 (3.90%), *Coprobacillus* - 2,283 (3.59%), *Thermosinus* - 2,161 (3.40%), *Drosophila* - 1,149 (1.81%), Pan - 738 (1.16%), *Cyanothece* - 644 (1.01%) |
| **5** | *Homo* - 37,654 (55.69%), *Macaca* - 6,452 (9.54%), *Canis* - 3,319 (4.91%), *Candidatus phytoplasma* - 3,217 (4.76%), *Propionibacterium* - 3,140 (4.64%), *Coprobacillus* - 2,195 (3.25%), *Alkaliphilus* - 1,933 (2.86%), *Thermosinus* - 1,374 (2.03%), *Clostridium* - 981 (1.45%), Pan - 817 (1.21%) |
| **6** | *Homo* - 32,261 (54.42%), *Finegoldia* - 9,184 (15.49%), *Macaca* - 4,048 (6.83%), *Canis* - 2,444 (4.12%), *Candidatus phytoplasma* - 1,830 (3.09%), *Coprobacillus* - 1,347 (2.27%), *Thermosinus* - 1,124 (1.90%), *Propionibacterium* - 564 (0.95%), *Drosophila* - 540 (0.91%), Pan - 528 (0.89%) |
| **7** | *Homo* - 30,247 (51.12%), *Macaca* - 5,696 (9.63%), *Candidatus phytoplasma* - 3,095 (5.23%), *Propionibacterium* - 2,449 (4.14%), *Canis* - 2,144 (3.62%), *Coprobacillus* - 2,100 (3.55%), *Bacteroides* - 2,038 (3.44%), *Aeromonas* - 1,970 (3.33%), *Clostridium* - 1,565 (2.65%), *Staphylococcus* - 1,208 (2.04%) |
| **8** | *Homo* - 26,710 (61.43%), *Macaca* - 4,365 (10.04%), *Candidatus phytoplasma* - 2,271 (5.22%), *Coprobacillus* - 1,819 (4.18%), *Canis* - 1,761 (4.05%), *Vibrio* - 1,708 (3.93%), *Drosophila* - 627 (1.44%), *Cyanothece* - 580 (1.33%), Pan - 517 (1.19%), *Propionibacterium* - 458 (1.05%) |
| **9** | *Homo* - 30,289 (56.49%), *Staphylococcus* - 5,932 (11.06%), *Macaca* - 4,960 (9.25%), *Candidatus phytoplasma* - 2,969 (5.54%), *Canis* - 2,151 (4.01%), *Coprobacillus* - 1,985 (3.70%), *Drosophila* - 757 (1.41%), *Cyanothece* - 614 (1.15%), Pan - 571 (1.06%), *Danio* - 224 (0.42%) |
| **10** | *Homo* - 24,897 (60.21%), *Macaca* - 4,669 (11.29%), *Canis* - 2,805 (6.78%), *Candidatus phytoplasma* - 2,235 (5.40%), *Coprobacillus* - 1,549 (3.75%), Pan - 598 (1.45%), *Vibrio* - 574 (1.39%), *Cyanothece* - 540 (1.31%), *Drosophila* - 516 (1.25%), *Pongo* - 198 (0.48%), *Staphylococcus* - 187 (0.45%) |
